# 溶剂萃取-高效液相色谱-四极杆飞行时间质谱法快速提取检测干燥恰特草中5种生物碱成分

**DOI:** 10.3724/SP.J.1123.2023.03009

**Published:** 2023-09-08

**Authors:** Hongfei SHI, Bopeng XU, Chengxin XU, Xiuqi ZHOU, Hongfu XU

**Affiliations:** 1.南京警察学院刑事科学技术学院, 江苏 南京 210023; 1. College of Criminal Science and Technology, Nanjing Police University, Nanjing 210023, China; 2.南宁市公安局特警支队, 广西 南宁 530022; 2. Nanning Municipal Public Security Bureau Special Police Detachment, Nanning 530022, China

**Keywords:** 高效液相色谱, 四极杆飞行时间质谱法, 甲卡西酮, 乙卡西酮, 精神活性成分, 恰特草, high performance liquid chromatography (HPLC), quadrupole time-of-flight mass spectrometry (Q-TOF MS), methcathinone, ethcathinone, psychoactive ingredients, khat

## Abstract

恰特草是一种常见的毒品原植物,卡西酮、去甲伪麻黄碱、去甲麻黄碱是其主要的精神活性成分。筛查发现恰特草中还存在具有较强精神活性作用的甲卡西酮和乙卡西酮成分。国内关于恰特草中生物碱成分的提取检测研究较少,国外也未见恰特草中甲卡西酮和乙卡西酮成分的研究。本研究通过优选提取溶剂种类、优化净化条件和色谱-质谱条件,利用0.05 mol/L的盐酸水溶液提取,二氯甲烷和1 mol/L的NaOH水溶液净化,再利用乙腈萃取,采用ZORBAX Eclipse Plus Phenyl-Hexyl色谱柱(100 mm×3.0 mm, 1.8 μm)分离,以0.1%甲酸水溶液-乙腈为流动相梯度洗脱,电喷雾双喷离子源(Dual AJS ESI)正离子模式下电离,四极杆飞行时间质谱仪目标离子采集模式(Target MS/MS)检测,外标法定量,建立了溶剂萃取-高效液相色谱-四极杆飞行时间质谱法提取检测干燥恰特草中5种生物碱成分的方法。结果表明,5种生物碱成分在各自的线性范围内线性关系良好,相关系数(*r*^2^)≥0.9976,检出限为0.08~0.75 μg/L,定量限为0.25~2.50 μg/L。2份生物碱含量差异较大的恰特草中5种生物碱成分的加标回收率为90.7%~105.2%,实验仪器精密度为0.5%~2.3%,日内方法精密度为1.0%~2.5%,日间方法精密度为1.3%~3.3%。该方法净化效果好,定量准确,灵敏度高,重复性好。利用该方法对国家林业局森林公安司法鉴定中心受理的15份恰特草相关案件中的检材进行检验,均检出5种生物碱成分,能够满足恰特草的理化检验鉴定要求,为恰特草中未知精神活性成分的发现和研究提供参考和指引。

## Abstract (en)

Khat is a common plant that grows primarily in Eastern Africa and the Arabian Peninsula. Cathinone, norpseudoephedrine, and norephedrine are the main psychoactive components of khat. Experimental studies have shown that red and green khat have similar cathinone contents, but green khat contains more norpseudoephedrine and norephedrine than red khat. Research indicates that Ethiopians believe that red khat has stronger psychoactive effects than green khat. Therefore, we speculated that other substances in red khat may enhance its psychoactive effects. Using the sampling method, we identified two other psychoactive components in khat: methcathinone and ethcathinone. At present, only a few studies on the extraction and detection of alkaloids from khat have been published in China, and no reports on the extraction and detection of methcathinone and ethcathinone from khat are available. In this study, we established an extraction and detection method for five alkaloids in dried khat using high performance liquid chromatography-quadrupole time-of-flight mass spectrometry (HPLC-Q-TOF MS). To establish the extraction method, we optimized the extraction solvent and process. The amounts of dichloromethane and sodium hydroxide added during the purification step were also optimized. To establish the detection method, we optimized the chromatographic and MS conditions. The final extraction and detection method was as follows: Dried khat powder (0.1 g) was loaded into a polypropylene centrifuge tube, added with 1 mL of 0.05 mol/L hydrochloride aqueous solution, and vortex-oscillated for 3 min for extraction. The sample was centrifuged at 10000 r/min for 3 min. Next, 600 μL of the supernatant was placed in a centrifuge tube, added with 1 mL of dichloromethane, shaken for 1 min, and centrifuged at 10000 r/min for 3 min. Subsequently, 300 μL of the supernatant was placed in a centrifuge tube, added with 80 μL of 1 mol/L sodium hydroxide aqueous solution, shaken for 1 min, and added with 1 mL of acetonitrile. Vortex oscillation was performed for 2 min to extract the sample, after which solid sodium chloride (0.4 g) was added to the mixture, followed by shaking for 1 min to separate the acetonitrile and aqueous phases. The mixture was then centrifuged at 10000 r/min for 3 min. Finally, the supernatant was collected and diluted for further testing. The five target analytes were separated on a ZORBAX Eclipse Plus Phenyl-Hexyl column (100 mm×3.0 mm, 1.8 μm) via gradient elution using 0.1% acetic acid aqueous solution and acetonitrile as mobile phases with a flow rate of 0.3 mL/min and column temperature of 30 ℃. The analytes were identified using the targeted MS/MS method under positive electrospray ionization mode and quantified using the external standard method. The five alkaloids showed good correlations (all correlation coefficients (*r*^2^)≥0.9976) with their respective linear ranges. The limits of detection were between 0.08 and 0.75 μg/L, and the limits of quantification were between 0.25 and 2.50 μg/L. The average recoveries of the five alkaloids from two plants with different alkaloid contents were between 90.7% and 105.2%. The intra-sample precision ranged from 0.5% to 2.3%, the intra-day precision was between 1.0% and 2.5%, and the inter-day precision was between 1.3% and 3.3%. Using the developed method, we extracted and analyzed 15 khat samples, and detected five alkaloids. This method enables rapid sample pretreatment and has high sensitivity, good stability, and suitable accuracy. Based on the above results, we conclude that the proposed method meets the inspection and identification requirements for khat. Thus, it can provide a valuable reference for the physical and chemical identification of khat and support for further studies on its psychoactive components.

恰特草(*Catha edulis*, khat)又名阿拉伯茶、巧茶,是无患子目、卫矛科、巧茶属常绿开花灌木,主要生长在非洲东部、阿拉伯半岛海拔1500~2500 m的地区^[1,2]^。埃塞俄比亚、也门等国家和地区吸食人数较多^[3,4]^,国内常出现在海关走私案件中。长期服用恰特草会造成抑郁症、口腔黏膜黑棘皮病^[5]^,并对心脏、神经等组织器官造成严重损伤^[6]^。2013年,国家食品药品监管总局、公安部、卫生计生委联合发布的《精神药品品种目录(2013年版)》中明确规定,将恰特草及其主要精神活性成分卡西酮(cathinone)列为第一类精神药品进行管制,去甲伪麻黄碱(norpseudoephedrine)列为第二类精神药品进行管制^[7]^,去甲麻黄碱(norephedrine)的精神活性较弱,未列入管制目录。恰特草的司法鉴定是打击恰特草走私犯罪的重要一环,新鲜恰特草可从形态、DNA、理化性质三方面进行检验,但目前恰特草走私主要采取伪装成茶叶或粉碎为粉末的方式,且干燥会使恰特草中的DNA发生降解,因此难以从形态和DNA角度认定^[8]^。卡西酮和去甲伪麻黄碱的含量是恰特草理化检验的重要指标,但由于卡西酮稳定性较差,其在运输和干燥过程中会不同程度地部分降解为去甲伪麻黄碱和去甲麻黄碱,为理化检验增加了不确定性。

由于实验材料获取困难,国内开展的恰特草理化检验研究较少,对于干燥恰特草中是否含有可检测含量的卡西酮成分,国内存在争议。国外对于恰特草中卡西酮、去甲伪麻黄碱、去甲麻黄碱的研究则较为全面,研究内容包括植物体内不同部位^[9⇓⇓⇓⇓-14]^、不同产地和品种的恰特草间^[10,13,15⇓-17]^的含量差异情况,生物碱含量随干燥和储存过程的变化情况^[11,18⇓⇓⇓⇓-23]^,在植物和人体内的代谢途径^[10,13,24⇓⇓⇓⇓-29]^及毒理学性质^[30⇓-32]^,提取检测方法等。相关研究中对于不同品种恰特草中生物碱成分的含量存在一定争议:埃塞俄比亚人对恰特草依照颜色分类,普遍认为红色恰特草药效更好,卡西酮含量更高,价格也更高^[16]^,但Krizevski等^[10]^的研究表明二者卡西酮含量相似,且绿色品种中去甲麻黄碱、去甲伪麻黄碱含量更高,由此推测恰特草中可能存在其他物质直接或间接地增强了红色品种恰特草的精神活性作用。

目前对于恰特草中卡西酮、去甲麻黄碱、去甲伪麻黄碱3种主要精神活性成分多采用气相色谱法^[12]^、气相色谱-红外光谱法^[23]^、气相色谱-质谱法^[9,10,18,21,33,34]^、薄层色谱法^[13]^、毛细管电泳法^[35]^、离子交换高效液相色谱法^[11]^、高效液相色谱法^[13,16,23,36,37]^、高效液相色谱-核磁共振联用法^[19,21]^、高效液相色谱-串联质谱法^[38,39]^进行检测。高效液相色谱-高分辨质谱法的研究较少,其能够通过质谱裂解规律和碎片离子精确质量数准确地对已知^[40]^、未知^[41,42]^化合物成分进行结构解析和物质确证,相对于高效液相色谱-低分辨质谱法,其对同分异构体和结构类似物有更强的分辨识别能力^[43]^;同时,高分辨质谱法对前端色谱分离要求较低,拥有更高的检测通量^[44,45]^,广泛应用于未知物分析、农药残留检测、代谢组学等领域。基于此,本研究团队利用高效液相色谱-四极杆飞行时间质谱法对恰特草中卡西酮的同系物进行筛查,发现恰特草中存在甲卡西酮(methcathinone)和乙卡西酮(ethcathinone)成分(相关确证内容将另文发表)。研究表明甲卡西酮和乙卡西酮均拥有较强的精神活性作用^[46]^,目前,甲卡西酮被列入第一类精神药品,乙卡西酮被列入《非药用类麻醉药品和精神药品管制品种增补目录》管制。

本文基于溶剂萃取-高效液相色谱-四极杆飞行时间质谱法建立了恰特草中卡西酮、去甲麻黄碱、去甲伪麻黄碱、甲卡西酮、乙卡西酮等5种生物碱成分的提取检测方法,为恰特草的理化检验鉴定及精神活性成分的研究提供参考。

## 1 实验部分

### 1.1 仪器、试剂与材料

Agilent 1290-6545高效液相色谱-四极杆飞行时间质谱仪,配电喷雾双喷离子源(Dual AJS ESI源)(美国Agilent公司); ME104E电子天平(瑞士Mettler-Toledo公司); Lab Dancer涡旋振荡仪(德国IKA公司); Pico17微量高速离心机(美国Thermo Fisher公司); Milli-Q纯水仪(美国Millipore公司)。

恰特草由国家林业局森林公安司法鉴定中心提供;卡西酮标准溶液(质量浓度为1000 μg/mL,纯度>99%)购自美国Cerilliant公司;去甲伪麻黄碱标准溶液(质量浓度为1000 μg/mL,纯度>99%)和甲卡西酮、乙卡西酮、去甲麻黄碱标准溶液(质量浓度为100 μg/mL,纯度>99%)购自天津阿尔塔科技有限公司。

浓盐酸、氢氧化钠、二氯甲烷、氯化钠(分析纯)购自南京化学试剂股份有限公司;甲醇、乙腈(色谱纯)购自美国Thermo Fisher公司。

### 1.2 标准溶液配制

单一标准储备液的配制:精密移取卡西酮、去甲伪麻黄碱标准溶液0.05 mL,去甲麻黄碱、甲卡西酮、乙卡西酮标准溶液各0.50 mL,分别置于5 mL容量瓶中,利用乙腈定容至刻度,混匀,制成质量浓度为10 mg/L的单一标准储备液,置于棕色试剂瓶,-20 ℃保存。

混合标准中间液的配制:精密移取5种生物碱成分的单一标准储备液各1 mL,置于同一10 mL容量瓶中,利用乙腈定容至刻度,混匀,制成质量浓度为1 mg/L的混合标准中间液,置于棕色试剂瓶,-20 ℃保存。

系列混合标准工作溶液的配制:利用0.1%甲酸水溶液将混合标准中间液稀释成质量浓度为1、5、10、25、50、75、100、150、200 μg/L的系列混合标准工作溶液。

### 1.3 实验条件

#### 1.3.1 样品前处理条件

精确称取100 mg粉碎后的干燥恰特草于2 mL聚丙烯离心管中,加入1 mL 0.05 mol/L的盐酸水溶液,涡旋3 min,以10000 r/min离心3 min;取上清液600 μL于2 mL离心管中,加入1 mL二氯甲烷,涡旋1 min,以10000 r/min离心3 min;取上清液300 μL于2 mL微量离心管中,加入80 μL 1 mol/L的氢氧化钠水溶液,涡旋1 min,加入1 mL乙腈,涡旋振荡2 min,加入400 mg NaCl固体,涡旋1 min,以10000 r/min离心3 min;取上清液,用0.1%甲酸水溶液稀释5倍、100倍、1000倍,分别对5种目标组分进行定量分析。

#### 1.3.2 色谱条件

色谱柱:ZORBAX Eclipse Plus Phenyl-Hexyl(100 mm×3.0 mm, 1.8 μm);柱温:30 ℃;流动相:A为0.1%甲酸水溶液,B为乙腈;进样量为5 μL;流速:0.3 mL/min;梯度洗脱程序:0~3.0 min, 5%B; 3.0~7.0 min, 5%B~40%B; 7.0~8.0 min, 40%B~60%B; 8.0~10.0 min, 60%B; 10.0~10.5 min, 60%B~5%B; 10.5~12.0 min, 5%B。

#### 1.3.3 质谱条件

离子源:Dual AJS ESI源;扫描方式:正离子全扫描;毛细管电压:4000 V;鞘气温度:350 ℃;鞘气流速:11 mL/min;干燥气温度:320 ℃;干燥气流速:8 mL/min;单极质谱全扫描模式(MS),一级质谱扫描范围*m/z* 100~1000,采集速率为5 spectra/s。目标离子采集模式(Target MS/MS),一级质谱扫描范围*m/z* 100~1000,采集速率为1 spectra/s,二级质谱扫描范围*m/z* 50~600,采集速率为2 spectra/s。数据采集时采用参比离子(C_5_H_4_N_4_,嘌呤,其准分子离子精确质量数为121.0509)和(C_18_H_18_O_6_, HP-0921,其准分子离子精确质量数为922.0098)进行实时质量校准。5种生物碱的保留时间、母离子精确质量数、子离子精确质量数、碎裂电压、碰撞能等见表1。

**表 1:** 5种生物碱的保留时间、母离子精确质量数、子离子精确质量数、碎裂电压、碰撞能

Compound	Retention time/min	Precursor ion (m/z)	Fragment ion (m/z)	Fragmentor/V	CE/eV
Norephedrine	5.32	152.1070	134.0964^*^	130	10
			117.0699		
Norpseudoephedrine	5.77	152.1070	134.0964^*^	130	10
			117.0699		
Cathinone	6.00	150.0913	117.0573^*^	140	26
			105.0699		
Methcathinone	6.41	164.1070	146.0964^*^	140	10
			131.0730		
Ethcathinone	6.75	178.1226	160.1121^*^	160	10
			132.0808		

*Quantitative ion.

## 2 结果与讨论

### 2.1 样品稀释溶剂的优化

采用乙腈、不同比例的乙腈-0.1%甲酸水溶液(8∶1、7∶2、6∶3、5∶4、4∶5、3∶6、2∶7、1∶8, v/v)、0.1%甲酸水溶液为稀释溶剂,将乙腈为溶剂的100 μL混合标准中间液稀释为1 mL 100 μg/L的混合标准溶液后进样分析。当使用乙腈、乙腈-0.1%甲酸水溶液(8∶1、7∶2、6∶3、5∶4、4∶5、3∶6, v/v)稀释混合标准中间液时,5种生物碱成分均存在溶剂效应(出现峰展宽、峰分叉现象),采用乙腈-0.1%甲酸水溶液(2∶7、1∶8, v/v)、0.1%甲酸水溶液为稀释溶剂时,无明显溶剂效应产生,因此选择0.1%甲酸水溶液为标准样品和实验样品的稀释溶剂。

### 2.2 色谱柱的优化

本文对比了3种色谱柱Eclipse Plus Phenyl-Hexyl (100 mm×3.0 mm, 1.8 μm)、Eclipse Plus C_18_ (100 mm×3.0 mm, 1.8 μm)、SB-C_18_ (50 mm×3.0 mm, 1.8 μm)对5种生物碱(质量浓度均为100 μg/L)的分离效果,结果见图1。SB-C_18_色谱柱的固定相采用不封端型键合方式,硅胶表面裸漏的硅羟基对碱性化合物会产生额外的吸附作用,造成色谱峰展宽、拖尾,不利于定性定量检测;Eclipse Plus C_18_和Eclipse Plus Phenyl-Hexyl色谱柱的固定相采用封端型的键合方式,色谱峰峰形良好;Eclipse Plus C_18_色谱柱的C_18_键合基团对5种生物碱成分的保留选择性小,无法将卡西酮和去甲伪麻黄碱完全分离;苯基键合色谱柱Eclipse Plus Phenyl-Hexyl通过*π-π*相互作用可以实现5种生物碱成分的基线分离,因此选择Eclipse Plus Phenyl-Hexyl色谱柱(100 mm×3.0 mm, 1.8 μm)进一步优化提取检测条件。

**图 1:**
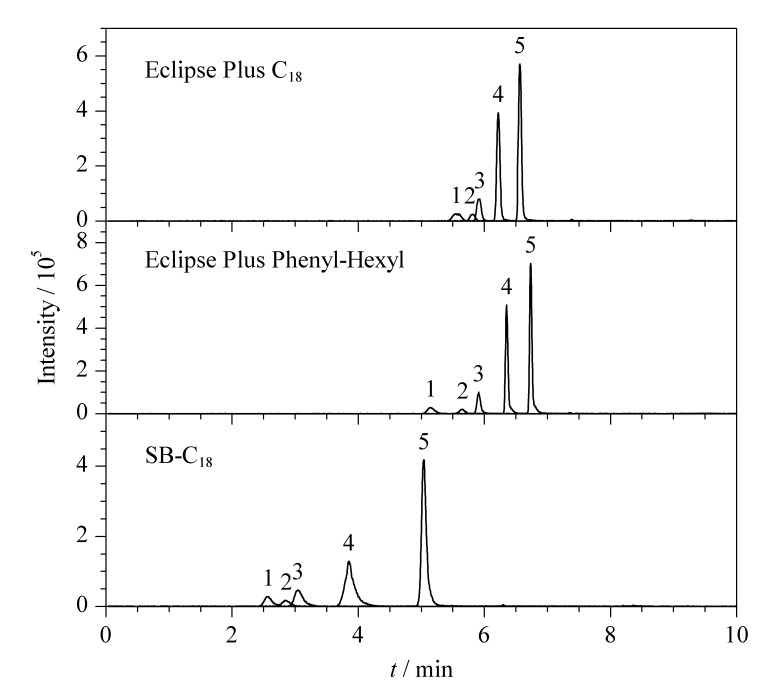
采用3种色谱柱时5种生物碱成分的提取离子色谱图

### 2.3 质谱条件优化

毛细管出口区域的碎裂电压对目标化合物准分子离子的响应有较强影响,过高或过低均不利于方法灵敏度的提升。过高时5种生物碱成分易发生源内裂解,中性丢失一分子H_2_O,过低时化合物准分子离子的离子传输效率降低。本研究在MS采集模式下,考察了碎裂电压在110~170 V范围内5种生物碱成分准分子离子峰的响应情况,确定去甲伪麻黄碱和去甲麻黄碱的最佳碎裂电压为130 V,卡西酮和甲卡西酮为140 V,乙卡西酮为160 V,结果见图2。

**图 2:**
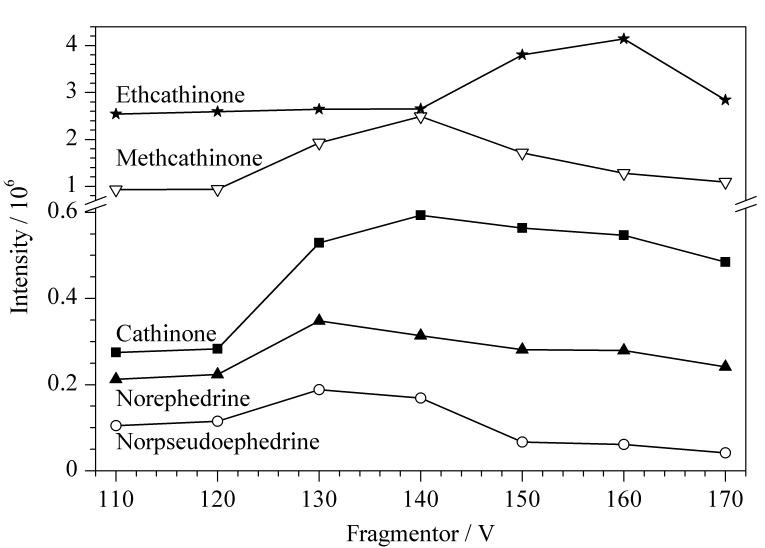
5种生物碱成分在不同碎裂电压下的提取离子色谱峰面积

在Target MS/MS采集模式下调整碰撞能,选取响应最强的子离子作为定量离子,选取响应次强的离子作为定性离子。优化碰撞能,使定量离子的响应达到最强,确定去甲麻黄碱、去加伪麻黄碱、甲卡西酮和乙卡西酮的最佳碰撞能为10 eV,卡西酮为26 eV。

### 2.4 样品提取条件的优化

本文以测定的恰特草中5种生物碱含量为评价标准,优化了提取溶剂和提取方法,并通过加标回收试验,评估了提取液的基质效应。

#### 2.4.1 样品提取溶剂和提取方法的优化

首先考察了7种溶剂(水、甲醇、乙腈、1%甲酸乙腈、0.10 mol/L的盐酸水溶液、乙腈-水(1∶1, v/v)、甲醇-水(1∶1, v/v))的提取效果。取100 mg干燥恰特草样品,加入1 mL提取溶剂振荡,上清液用0.1%甲酸水溶液稀释,参照1.3节的色谱-质谱条件检测。结果显示,0.10 moL/L的盐酸水溶液和乙腈-水(1∶1, v/v)提取效果较好,见图3。

**图 3:**
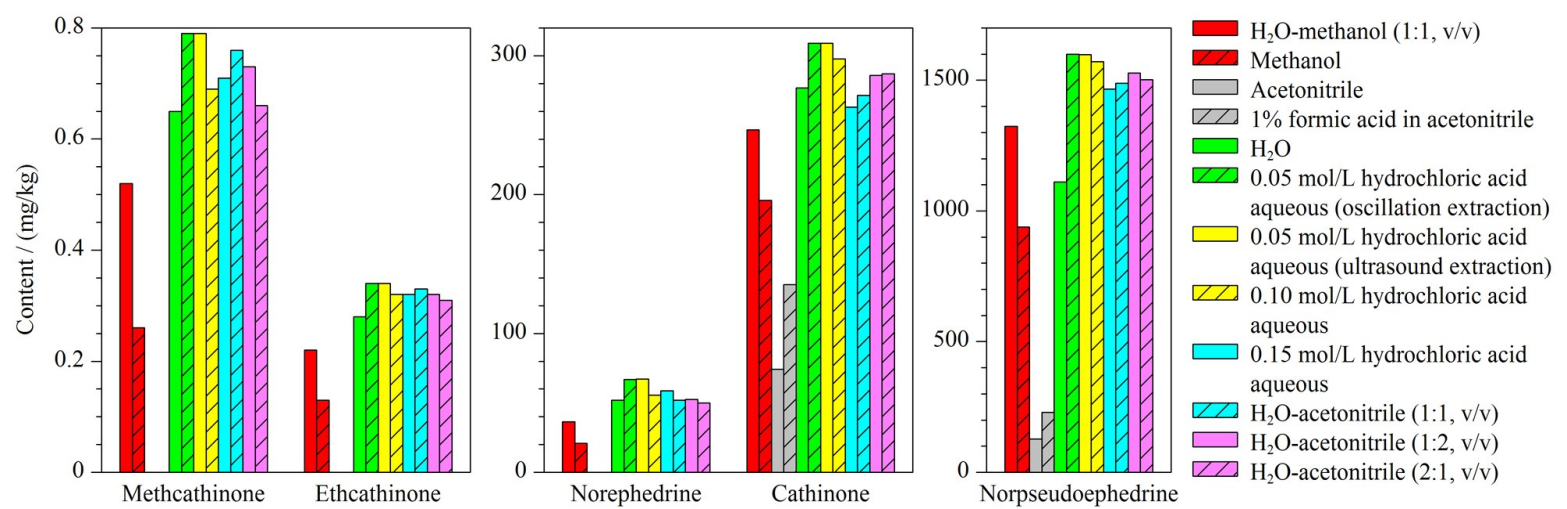
11种提取溶剂和2种提取方法提取的恰特草中5种生物碱成分的测定值

为进一步考察不同浓度盐酸水溶液及不同比例乙腈-水的提取效果,选取0.05 mol/L和0.15 mol/L的盐酸水溶液、乙腈-水(2∶1、1∶2, v/v)4种提取溶剂,参照上述方法提取检测。结果显示,0.05 mol/L的盐酸水溶液提取效果最佳,见图3。

考察了使用0.05 mol/L的盐酸水溶液分别采用振荡提取3 min和30 ℃超声提取15 min两种提取方法,二者提取效果无显著差异,结果见图3,因此提取方法选择用时短、简单快捷的振荡提取法。

#### 2.4.2 提取液原液的基质影响考察

取6份干燥恰特草样品,每份100 mg,其中3份样品的加标水平为去甲麻黄碱20 mg/kg、去甲伪麻黄碱300 mg/kg、卡西酮200 mg/kg、甲卡西酮0.8 mg/kg、乙卡西酮0.4 mg/kg,用1 mL 0.05 mol/L的盐酸水溶液对3份恰特草加标样品及3份未加标样品进行提取。取300 μL上清液,加入700 μL乙腈,混匀,用0.1%甲酸水溶液稀释5倍、10倍、100倍、500倍,参照1.3节的色谱-质谱条件分别检测甲卡西酮、乙卡西酮、去甲麻黄碱、卡西酮和去甲伪麻黄碱。

加标回收率通过如下公式计算:加标回收率=(加标样品生物碱测定值-未加标样品生物碱测定值)/标准添加量×100%。由于稀释倍数不同,5种化合物加标回收率受基质效应的影响存在差异,5种生物碱的加标回收率为120.6%~146.8%。同时提取液中有2种其他卡西酮类似物存在,其二级质谱碎片离子的精确质量数和相对丰度与卡西酮相同,但色谱保留时间存在较大差异,结果见图4。

**图 4:**
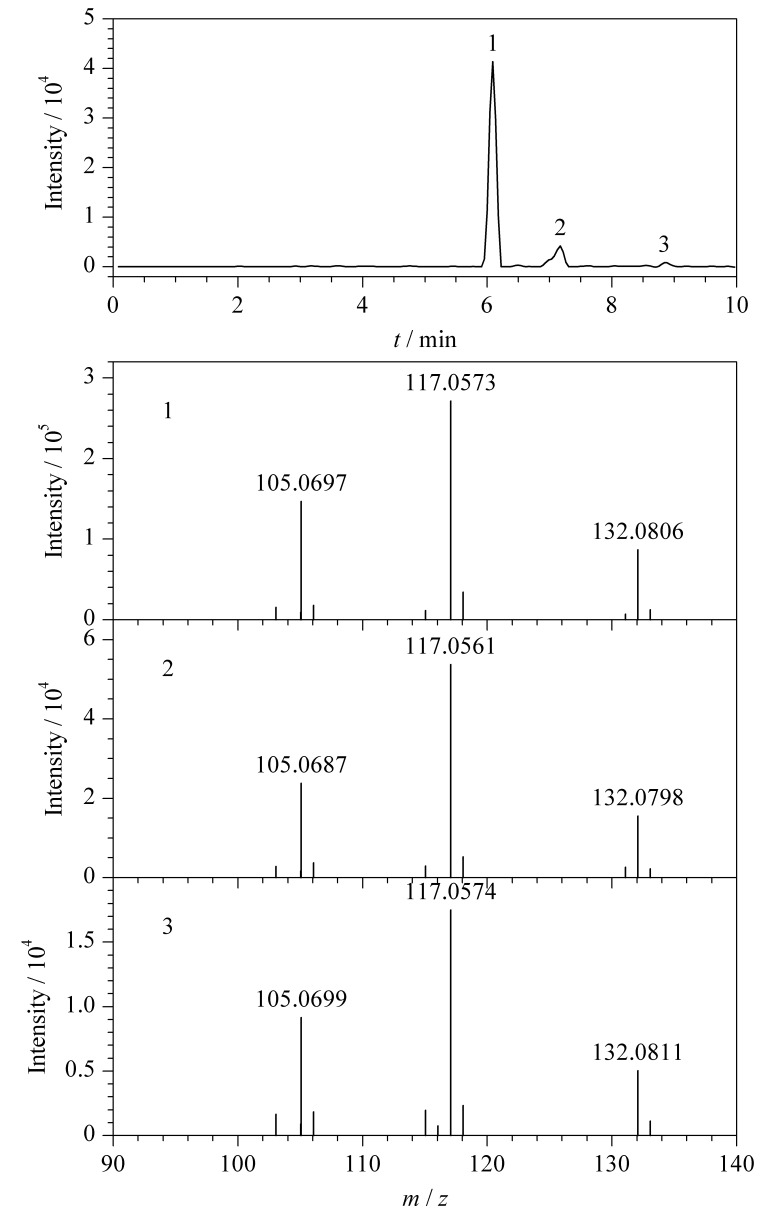
净化前恰特草样品中卡西酮二级质谱分析特征离子对 (150.0913/117.0573)的提取离子色谱图及碰撞能为25 eV下卡西酮和两种卡西酮类似物的二级质谱图

综上,为去除杂质成分,减少对色谱柱和离子源的污染,降低基质效应,提升定量分析的准确性,消除卡西酮类似物对恰特草中卡西酮成分定性定量分析的干扰,需建立并优化提取液的净化方法。

### 2.5 样品净化条件的优化

5种生物碱成分均为水溶性生物碱^[46]^,在酸性条件下为离子状态,易溶于水,不溶于非极性有机溶剂,在碱性条件下为分子状态,易溶于极性有机溶剂。实验利用生物碱的溶解特性通过溶剂萃取法实现恰特草提取液的净化。

#### 2.5.1 NaOH溶液添加量的优化

实验中用1 mL二氯甲烷净化提取液,去除色素、甾醇和其他极性较弱的小分子化合物;加入1 mol/L NaOH溶液,调节pH值,使5种生物碱成分转变为分子状态,并使提取液中的大分子蛋白质发生变性,生成絮状沉淀。本文考察了不同体积(0、30、40、50、60、70、80、90、100 μL)的1 mol/L NaOH溶液对测定恰特草中5种生物碱含量的影响,结果显示,NaOH溶液的最佳添加量为80 μL,见图5。净化完成后用乙腈萃取提取液,加入固体NaCl使乙腈和水分层,取上清液,利用0.1%甲酸水溶液稀释,参照1.3节中的色谱-质谱条件检测,结果显示,该方法消除了卡西酮类似物对恰特草中卡西酮成分定性定量分析的干扰,见图6。

**图 5:**
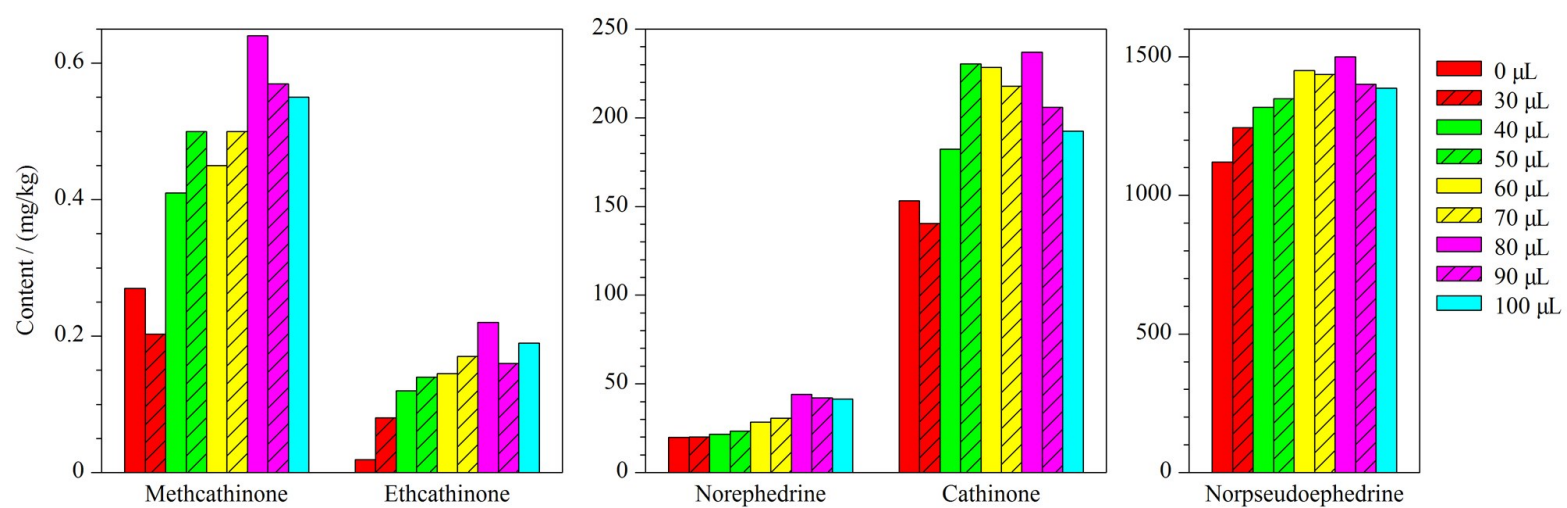
不同体积1 mol/L NaOH溶液净化后恰特草中5种生物碱成分的测定值

**图 6:**
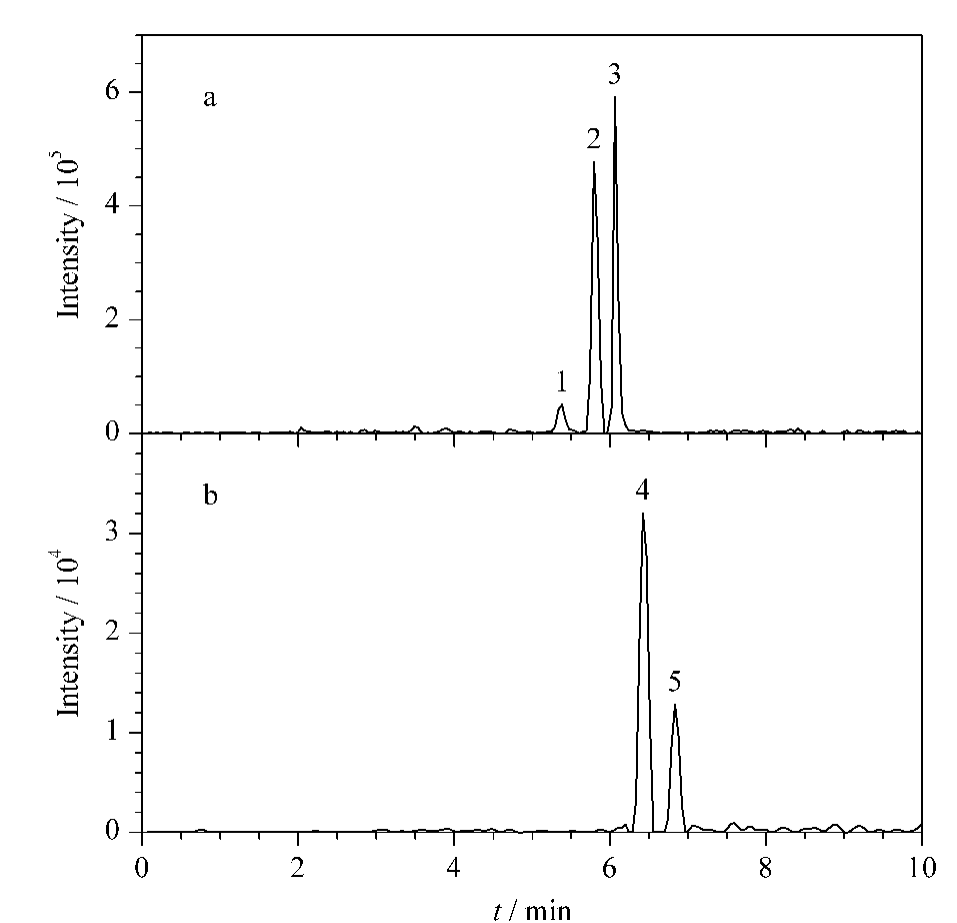
净化后的恰特草样品中5种生物碱的提取离子色谱图

#### 2.5.2 净化对生物碱回收率及样品基质效应的影响

净化后恰特草中5种生物碱成分的测定值低于净化前,为评价净化效果,明确测定值降低的原因,实验探究了净化过程对生物碱回收率及样品基质效应的影响。

净化过程对生物碱回收率的影响:用0.05 mol/L的盐酸水溶液配制3份1 mL质量浓度为600 μg/L的5种生物碱混合标准溶液。参照1.3节中条件提取检测,5种生物碱回收率为98.1%~104.3%。

净化过程对样品基质效应的影响:制备6份恰特草加标样品和6份未加标样品,取3份恰特草加标样品和3份未加标样品参照1.3节中的方法提取检测,5种生物碱成分的加标回收率为92.8%~108.6%。另取3份恰特草加标样品和3份未加标样品,除不采用二氯甲烷净化外,参照1.3节中的条件提取检测,5种生物碱成分的加标回收率为115.4%~129.6%。

结果显示,净化过程对5种生物碱的回收率无显著影响。使用NaOH溶液除去大分子蛋白质,样品仍存在较强的基质效应,使用二氯甲烷能有效去除部分色素、甾醇和其他极性较弱的小分子化合物,与NaOH溶液同时使用,能有效减弱样品的基质效应。

### 2.6 方法学验证

#### 2.6.1 线性范围、检出限及定量限

将1、5、10、20、25、50、75、100、150、200 μg/L系列混合标准工作溶液参照1.3节的色谱-质谱条件检测,以定量离子色谱峰面积为纵轴(*y*),化合物的质量浓度为横轴(*x*),利用Agilent MassHunter Quantitative Analysis B.07.01 (for Q-TOF)软件绘制工作曲线,依据恰特草中5种生物碱的含量,选取合适的线性范围,计算相关系数(*r*^2^),5种生物碱的相关系数均≥0.9976。

利用Agilent MassHunter Qualitative Analysis B.07.00软件以peak to peak的方式计算信噪比,以目标化合物的信噪比等于3(*S/N*=3)确定该化合物的检出限,以*S/N*=10确定该化合物的定量限。检出限为0.08~0.75 μg/L,定量限为0.25~2.50 μg/L,满足恰特草的检验鉴定要求。5种生物碱成分的线性回归方程、线性范围、相关系数、检出限与定量限见表2。

**表 2:** 5种生物碱成分的线性回归方程、线性范围、相关系数、检出限与定量限

Compound	Regression equation	Linear range/(μg/L)	r^2^	LOD/(μg/L)	LOQ/(μg/L)
Norephedrine	y=4.71×10^3^x+2.74×10^4^	10-150	0.9976	0.75	2.50
Norpseudoephedrine	y=3.18×10^3^x+1.96×10^4^	10-150	0.9978	0.75	2.50
Cathinone	y=3.34×10^3^x+2.09×10^4^	10-150	0.9995	0.34	1.16
Methcathinone	y=1.56×10^4^x+1.96×10^2^	1-25	0.9994	0.08	0.25
Ethcathinone	y=9.15×10^3^x-1.53×10^2^	1-25	0.9992	0.17	0.55

y: peak area of the quantitative ion; x: mass concentration, μg/L.

#### 2.6.2 加标回收率与方法精密度

在方法实际应用过程中,发现不同恰特草中生物碱成分的含量存在差异。因此另取A、B两份生物碱含量差异较大的干燥恰特草样品评价加标回收率和方法精密度。

取12份A组干燥恰特草样品,每份100 mg,其中6份加标水平为去甲麻黄碱20.0 mg/kg、去甲伪麻黄碱300.0 mg/kg、卡西酮200.0 mg/kg、甲卡西酮0.8 mg/kg、乙卡西酮0.4 mg/kg;取12份B组干燥恰特草样品,每份100 mg,其中6份加标水平为去甲麻黄碱20.0 mg/kg、去甲伪麻黄碱500.0 mg/kg、卡西酮30.0 mg/kg、甲卡西酮0.2 mg/kg、乙卡西酮0.2 mg/kg。参照1.3节中的条件提取检测。参照公式(1)计算恰特草中生物碱的含量,由于前处理过程中1 mL提取液中仅有300 μL提取液用于进一步净化检测,因此公式中需对恰特草质量乘以系数0.3,计算加标回收率,结果见表3。5种生物碱成分的添加回收率为90.7%~105.2%,该方法拥有良好的提取效果,且净化过程能有效减弱样品基质效应,满足恰特草中生物碱成分的提取检测要求。


(1) $\omega=\frac{V D C}{0.3 M}$

式中:*ω*为生物碱含量(mg/kg); *V*为提取溶液体积(L); *D*为上清液稀释倍数;*C*为利用标准曲线计算的稀释液中生物碱成分的质量浓度(μg/L); *M*为干燥恰特草质量(g)。

**表 3:** 两份恰特草样品中5种生物碱成分的加标回收率及其相对标准偏差(n=6)

Compound	Sample A					Sample B			
	Background/ (mg/kg)	Added/ (mg/kg)	Recovery/ %	RSD/ %		Background/ (mg/kg)	Added/ (mg/kg)	Recovery/ %	RSD/ %
Norephedrine	46.4	20.0	105.2	2.9		73.8	20.0	96.7	1.8
Norpseudoephedrine	1578.5	300.0	98.7	1.4		1716.8	500.0	102.2	2.0
Cathinone	266.3	200.0	103.2	2.0		40.2	30.0	92.8	1.1
Methcathinone	0.6	0.8	97.0	0.3		0.1	0.2	90.7	0.9
Ethcathinone	0.3	0.4	99.3	1.0		0.1	0.2	91.6	0.4

仪器精密度实验:对同一瓶提取液6次连续进样,计算测定结果的平均值及RSD;日内方法精密度实验:一天内不同时间对A、B两份干燥恰特草各重复6次实验;日间方法精密度实验:由3名实验员在6天内重复实验6次。实验仪器精密度为0.5%~2.3%,日内方法精密度为1.0%~2.5%,日间方法精密度为1.3%~3.3%,方法具有良好的稳定性和重复性,见表4。

**表 4:** 两份恰特草样品中5种生物碱的含量及实验仪器精密度、日内方法精密度、日间方法精密度

Compound	Contents in sample A/(mg/kg) (RSD/%)				Contents in sample B/(mg/kg) (RSD/%)		
	Intra-sample (n=6)	Intra-day (n=6)	Inter-day (n=6)		Intra-sample (n=6)	Intra-day (n=6)	Inter-day (n=6)
Norephedrine	47.9 (2.3)	47.6 (2.4)	45.2 (3.2)		75.9 (2.2)	75.2 (2.5)	76.8 (3.3)
Norpseudoephedrine	1575.3 (1.5)	1589.3 (2.0)	1588.2 (1.9)		1719.2 (2.3)	1721.4 (2.4)	1720.4 (2.3)
Cathinone	267.2 (1.8)	264.5 (1.0)	269.8 (2.2)		40.5 (0.7)	40.9 (1.6)	39.6 (1.9)
Methcathinone	0.6 (0.6)	0.6 (1.5)	0.6 (2.0)		0.1 (1.4)	0.1 (1.5)	0.1 (2.4)
Ethcathinone	0.3 (0.9)	0.3 (1.0)	0.3 (1.3)		0.1 (0.5)	0.1 (1.5)	0.1 (1.7)

### 2.7 实际样品检验

为检验提取检测方法和检测结果的可靠性,参照1.3节中的提取检测方法对国家林业局森林公安司法鉴定中心受理的15份干燥恰特草检材进行检测,15份干燥恰特草检材均检出去甲麻黄碱、去甲伪麻黄碱、卡西酮、甲卡西酮、乙卡西酮5种生物碱成分,说明该方法适用于干燥恰特草中生物碱成分的检测。

## 3 结论

本研究基于溶剂萃取-高效液相色谱-四极杆飞行时间质谱法建立了干燥恰特草中卡西酮等5种生物碱成分的提取检测方法。通过优选提取溶剂种类、优化净化条件和色谱-质谱条件,除去了提取液中的杂质成分,减少了样品分析对离子源和色谱柱的污染,减弱了样品基质效应,实现了5种生物碱的同时检测。经实际样品分析验证,该方法净化效果好,定量准确,灵敏度高,重复性好,能够满足恰特草的理化检验鉴定要求,并确认去甲麻黄碱、去甲伪麻黄碱、卡西酮、甲卡西酮和乙卡西酮5种精神活性成分普遍存在于干燥恰特草中,为恰特草的理化检验鉴定提供参考,并为恰特草中未知精神活性成分的发现和研究提供指引。
